# P-805. Duration of Intravenous Acyclovir in Adults and Children with Suspected Herpes Simplex Virus Encephalitis at an Institution with the BioFire FilmArray Meningitis/Encephalitis Panel

**DOI:** 10.1093/ofid/ofaf695.1014

**Published:** 2026-01-11

**Authors:** David A Gutierrez, Emily Kirkpatrick, Travis J Carlson

**Affiliations:** University Health System, San Antonio, TX; University Health, San Antonio, Texas; The University of Texas at Austin College of Pharmacy, San Antonio, Texas

## Abstract

**Background:**

Herpes simplex encephalitis (HSE) is a rare infection with a high mortality rate if left untreated. Polymerase chain reaction (PCR) testing of cerebrospinal fluid (CSF) is the diagnostic gold standard, but false negatives can occur. Clinical guidelines recommend a repeat lumbar puncture for additional CSF testing when suspicion for HSE remains high. However, confirmatory re-testing of initial CSF samples through third-party laboratories has been observed in clinical practice. This practice may prolong empiric intravenous acyclovir (IV ACV) therapy.

**Methods:**

This single-center retrospective cohort study included patients admitted to University Health in San Antonio, Texas, between July 1, 2020 and November 30, 2025, who received IV ACV and had CSF tested using the BioFire FilmArray Meningitis/Encephalitis Panel (BioFire ME Panel). All HSV-positive patients were included. Due to cohort size, HSV-negative adult patients were randomly sampled to match the number of HSV-negative pediatric patients. The primary outcome was IV ACV duration in hours following the BioFire ME Panel result.

**Results:**

Among 217 patients, 12 adults and 1 pediatric patients had an HSV-positive BioFire ME Panel result. The median (interquartile range) IV ACV duration after Biofire ME Panel result was 115.2 (65.5 – 210.7) hours for HSV-positive patients versus 18.1 (4.5 – 47.2) hours for HSV-negative patients (P = 0.0002). Confirmatory re-testing at a third-party laboratory was observed in 22/102 (21.6%) pediatric and 3/115 (2.6%) adult patients, but none of these tests were positive. In the pediatric only subgroup, patients with a negative Biofire ME Panel result who underwent third-party confirmatory testing received IV ACV for a median of 79.8 (36.2 - 137.0) hours after the initial result compared to 25.4 (7.8 - 51.2) hours in patients without confirmatory testing (P < 0.0001).

**Conclusion:**

Third-party PCR re-testing did not identify any false-negative BioFire ME Panel results and was associated with prolonged IV ACV use. Diagnostic stewardship initiatives targeting BioFire ME Panel utilization could reduce unnecessary testing, excessive antiviral exposure, associated costs, and treatment risks.

**Disclosures:**

Travis J. Carlson, PharmD, BCIDP, Aimmune Therapeutics, Inc.: Speaker bureau

